# Manual of Dental Surgery and Pathology

**Published:** 1882-06

**Authors:** 


### BIBLIOGRAPHICAL.

*Manual of Dental Surgery and Pathology.* By Alfred Coleman, L. R. C. P., etc., etc., etc. Thoroughly revised and adapted to the use of American Students, Practitioners, by Thomas C. Stellwagen, M. A., M. D., D. D. S. Publishers: Henry C. Lea's Son & Co., Philadelphia.

The reviser deserves the thanks of the profession for the labor he has bestowed on this work to adapt it to the wants of the American practitioner and student. Twenty-three Chapters embrace First and Second Dentition, Injuries of the Teeth, Dental Caries, Description of Instruments, Treatment of Dental Caries, Periodentitis Necrosis, Artificial Crowns, Extraction. Anæsthesia, Replantation and Transplantation of Teeth, Diseases of the Gums, Jaws, Antrum, etc., etc.

Like all English works on Operative Dentistry, its chief fault is a failure to fully describe some very important operations; such as restoration of contour, use of the electro-magnetic mallet, etc., etc.

The article on pivot crowns is excellent so far as it describes a limited number of modes of application, but there are others of value which have been altogether omitted. This Chapter is the work of the American reviser, and is well written and comprehensive. The revisor's method of "Capping" by means of the chips and powder cut from the healthy dentine of the tooth being treated, is given in full and his preference for such a protective covering clearly shown. The use of arsenious acid as an antiseptic in dead teeth is not a wise recommendation in our opinion, and should be resorted to with great caution, if at all. In the diagnosis of diseases of the periosteum, the work lacks the conciseness so necessary in classification of causes, &c., which should form one of the leading features in all descriptions of pathological conditions. We are aware of the difficulty of revising a work and at the same time preserving the identity of the author; hence Dr. Stellwagen deserves great credit for the manner in which he has arranged and annotated this publication. We have no doubt but that the reviser would have found it an easied task to have written a work entirely his own. It will prove, however, a valuable addition to dental literature, and is well worth some study. The neat and handsome style in which it is issued is worthy of the well known publishing house from whence it comes.

